# Benefits of inspiratory muscle training therapy in institutionalized adult people with cerebral palsy: A double‐blind randomized controlled trial

**DOI:** 10.1002/brb3.70044

**Published:** 2024-09-18

**Authors:** Carlos Martin‐Sanchez, Fausto Jose Barbero‐Iglesias, Victor Amor‐Esteban, Marta Martin‐Sanchez, Ana Maria Martin‐Nogueras

**Affiliations:** ^1^ Nursing and Physiotherapy Department University of Salamanca Salamanca Spain; ^2^ NEUROUSAL Research Group (Investigation in Neurorehabilitation) Salamanca Spain; ^3^ Statistics Department University of Salamanca Salamanca Spain; ^4^ Nursing Department Salamanca University Clinical Hospital Salamanca Spain

**Keywords:** aging, cerebral palsy, muscle strength, older adults, physical therapy

## Abstract

**Background:**

Respiratory health problems are one of the main causes of morbidity and mortality in adult people with cerebral palsy (CP). The influence of respiratory muscle training has not yet been studied in this population group. The objective of the study was to evaluate and compare the efficacy of two protocols with inspiratory muscle training (IMT), low intensity and high intensity, to improve respiratory strength and pulmonary function in adults with CP.

**Methods:**

The study was a controlled, randomized, double‐blind trial with allocation concealment. Twenty‐seven institutionalized CP patients were recruited and randomly distributed in the high‐intensity training group (HIT) or low‐intensity training group (LIT). Over 8 weeks, an IMT protocol was followed 5 days/week, 10 series of 1 min with 1 min rest between them. HIT trained with a load of 40% of the maximum inspiratory pressure (MIP) and LIT with 20%. Respiratory strength and pulmonary function were evaluated.

**Results:**

After IMT intervention, MIP, maximum expiratory pressure, forced expiratory volume in 1 s (FEV_1_) and peak expiratory flow increased in both groups; in HIT 29%, 19%, 13%, and 8%, respectively (*p* = 0.000, *p* = 0.000, *p* = 0.002, *p* = 0.001) and in LIT 17%, 7%, 3%, and 4%, respectively (*p* = 0.000, *p* = 0.000, *p* = 0.049, *p* = 0.113). All the improvements were significantly higher in HIT than in LIT.

**Conclusion:**

Inspiratory muscle training improved respiratory muscle strength and pulmonary function in adults with CP. Training with a 40% MIP load improved all the evaluated parameters and was the most effective treatment for adults with CP.

## Graphical abstract

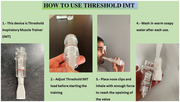

## 1 INTRODUCTION

Life expectancy is increasing, which is causing an aging of the world's inhabitants. All countries are experiencing an aging population growth. Within 30 years, 16% of people will be over 65 years old, 6% more than in 2022. In addition, according to United Nations forecasts, there will be twice as many people over 65 as children under 5 (United Nations, Department of Economics & Social Affairs, Population Division, 2022).

Cerebral palsy (CP), which occurs in one in 500 new live births, has multiple causes resulting in brain injury that affects movement, posture, and balance (Vitrikas et al., 2020). CP is a nonprogressive disorder; it does not get worse when children grow up (Mudge et al., 2016). However, as CP patients get older, the wear and tear of living with CP can extend symptoms and begin to cause other physical difficulties (Morgan & McGinley, 2018). Life expectancy has been increasing in people with CP in recent years (You Gyoung et al., 2019), and adult people may experience an increasing impact of their disability on daily life, which will affect their interpersonal relationships, autonomy, and quality of life (van Heijningen et al., 2021).

Respiratory disease is the most common cause of mortality, morbidity, and poor quality of life in the most severely affected children (Marpole et al., 2020). Adults with CP have a high risk of health problems that affect the respiratory system. Aspiration of feed or secretions, reduced functional lung volume, and loss of respiratory muscle strength (muscle weakness) are frequent complications in these patients (National Guideline Alliance (UK), 2019). These parameters are decreased in adult patients with CP by more than 50% compared with the reference data for a healthy population. Scoliotic deformity of the spine causes additional restriction of respiratory function. In addition, the decrease in chest expansion implies an alteration in respiratory mechanics that can affect the oxygen supply in some cases (Campbell et al., 2003; Ezeufwu et al., 2013; Lampe et al., 2014). Accordingly, inspiratory muscle training (IMT) is postulated as a treatment to combat the adverse effects suffered by patients with CP in the respiratory system (Keles et al., 2018). The effectiveness of IMT in improving these symptoms has been previously demonstrated in other population groups as elderly people (Martin‐Sanchez et al., 2021; Seixas et al., 2020), multiple sclerosis patients (Huang et al., 2020; Martin‐Sanchez et al., 2020), or COPD patients (Figueiredo et al., 2020).

Most of the existing information for grading training load with the inspiratory muscle training recommends working at 30% of maximum inspiratory pressure (MIP), including the manufacturer's recommendation. In population with severe disabilities and specifically in people with cerebral palsy, the workloads used range between 5% and 30% of MIP (Bhuvaneshwari & Suriliraj, 2021; Keles et al., 2018; Varol‐Kepenek et al., 2021). Recent studies in other population groups have used loads of 50% MIP, so the best training load is not yet clear (Abodonya et al., 2021; Craighead et al., 2021; McNarry et al., 2022).

Based on this information and the total absence of respiratory studies in adult people with cerebral palsy, the objective of this clinical trial was to assess and compare the usefulness of two training programs with IMT, high loads and low loads, to improve pulmonary function and respiratory strength in people with CP.

## 2 MATERIALS AND METHODS

### 2.1 Study design

The study was a controlled, randomized, double‐blind trial with allocation concealment. The study protocol was approved by the Bioethics Committee of the University of Salamanca (number of registry 678, October 6, 2021).

The study was registered in the clinical trials database of the United States National Library of Medicine (www.clinicaltrials.gov) with the number of registration NCT04915170.

### 2.2 Participants

**FIGURE 1:**
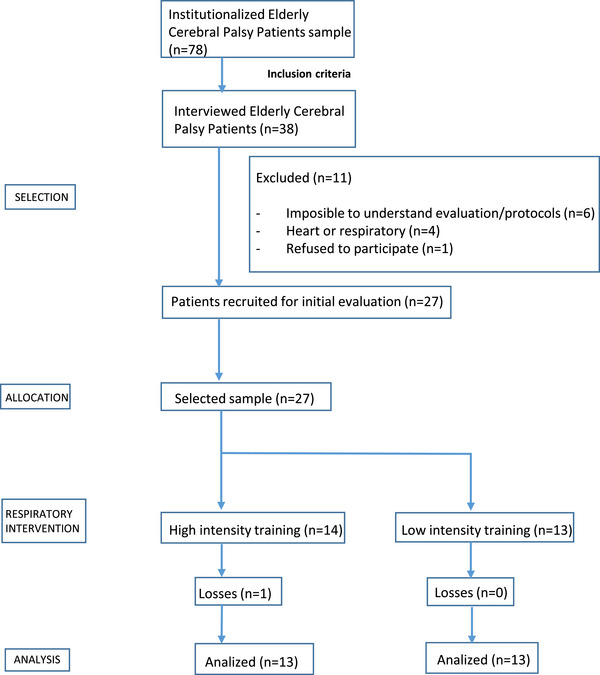
Flowchart of participants.

The experimental phase of this clinical trial was carried out between June and August 2022, 38 adult people with CP were selected to be part of the study, and 27 individuals were voluntarily recruited to participate in respiratory training and randomly distributed in the high‐intensity training group (HIT) or low‐intensity training group (LIT) (Figure 1).

Institutionalized people with cerebral palsy between 35 and 64 years were included, they were all members of ASPACE Salamanca. Gross motor function classification system (GMFCS) and mini‐mental state examination (MMSE) were evaluated at the beginning of the study. GMFCS: 3.66 ± 0.90; level I (*n* = 0), level II (*n* = 3), level III (*n* = 8), level IV (*n* = 11), level V (*n* = 5). MMSE: 17.96 ± 3.08.

Exclusion criteria: the presence of respiratory disease in the previous month, inability to understand assessment tests or intervention, hemodynamic alterations (heart rate > 150 beats per minute (bpm), systolic blood pressure > 140 millimeters mercury (mmHg), or diastolic blood pressure > 90 mmHg).

The sample size was determined by sampling calculation done from collected during a pilot study with 10 volunteers, which established a sample of 10 individuals for each group to detect differences of 12 cmH_2_O for MIP, considering a power of 80%, security 95%.

### 2.3 Procedures and measures of outcomes

Adult people with cerebral palsy were randomly allocated, via computerized random assignment, to either a high‐intensity intervention group or a low‐intensity intervention group. The professional who collected the data and the participants were unaware of the group assignment. Before and after inspiratory muscle training respiratory muscle strength and pulmonary function were evaluated.

### 2.4 Primary outcome

The primary outcome was respiratory muscle strength. It was measured using the MIP and the maximum expiratory pressure (MEP) with a pressure measurer (Elka PM‐15, Laboliser S.A.), from residual volume and total lung capacity, severally. Each measure was expressed in millibars and turned into centimeter of water (cm H_2_O; 1 mbar = 1.01973 cm H_2_O), reference unit, according to recommendations of the American Thoracic Society/European Respiratory Society (ATS/ERS; American Thoracic Society/European Respiratory Society, 2002). Each test was repeated three times or until two valid results were achieved (a difference of less than 5%). A rest time of 1 min was respected between efforts to prevent short‐term respiratory muscle fatigue. The highest value was selected. MIP was evaluated every 2 weeks to gradually increase the load of the device training.

### 2.5 Secondary outcome

The secondary outcome was pulmonary function, collecting forced expiratory volume in 1 s (FEV_1_) and peak expiratory flow (PEF) data. It was measured using the peak flow device (Asma‐1, Vitalograph Ltd) that expresses the results of FEV_1_ in liters (L) and PEF in liters per minute (L/min), according to the guidelines of the ATS/ERS (American Thoracic Society/European Respiratory Society, 2002).

### 2.6 Experimental protocol

Respiratory training performed through IMT was carried out with a pressure threshold device (Threshold IMT, Philips‐Respironics). Threshold IMT offers a constant and specific pressure for strength and endurance training of the respiratory muscles, regardless of the strength or speed with which the patient breathes. A flow‐independent one‐way valve ensures constant resistance and allows you to specifically adjust workload (in cmH_2_O). The training must be supervised by a healthcare professional. During the inspiration, a spring‐loaded valve resists to stimulate the training of the respiratory muscles. Before training began, the participants and primary caregivers completed one‐session familiarization with a specialist to know the operation of the device.

Adult people with cerebral palsy carried out the training program for 8 weeks, 1 session every day, 5 days a week. The participants performed 10 series of 1 min with 1‐min rest between them.

The HIT received IMT at 40% of MIP, the training load was set each 2 weeks to keep 40% of MIP. The LIT received IMT at 20% of MIP, following the same rules as HIT.

The training protocols of the two groups were developed by a specialist in respiratory therapy and all sessions with IMT were supervised by their main caregiver, therefore, adherence to the program was controlled, as well as possible unwanted effects (increased fatigue, breathing problems, dizziness, or sickness).

During the intervention period, both groups continued with their usual activity receiving physiotherapy treatment for 45 min per day, 2 days per week. No participants received any other treatments.

### 2.7 Statistical analysis

The characteristics of the sample were statistically expressed as mean and standard deviation (SD). The normality and homogeneity of the sample were assessed using the Shapiro–Wilk test. Parametric tests were used to compare results when a normal distribution was found. *T*‐test, Mann–Whitney *U*‐test, and Wilcoxon rank‐sum test were applied to analyze independent and paired samples, as necessary. HJ‐Biplot was used as a multivariate analysis technique to represent the individuals, the study data, and the interrelations in a reduced plane.

*T*‐test and Mann–Whitney test were used to compare the results and the effect size.

HJ‐Biplot belongs to Biplot methods and allows the study data obtained from the sample to be represented jointly in a multivariate scatterplot. It is very useful to express interrelationships between the different subjects of the study, representing them as marks on a plane. The position of the points in the graph indicates similarity or difference between participants, two points very close together confirm similar characteristics in the analyzed values. The angles between the vectors indicate direct relationships (acute angles), independence (right angles), or inverse relationships (obtuse angles). It is a statistical procedure to observe the study results clearly and simply.

## 3 RESULTS

Thirty‐eight adults with CP were selected as the study sample, of which 11 were excluded. Twenty‐seven were randomly assigned to HIT (*n* = 14) or LIT (*n* = 13). A caregiver helped the patients during the sessions, managing to complete 100% of them. No adverse effects were reported during the treatments.

The initial characteristics of the participants are expressed in Table 1. Both groups presented similar values at the beginning of the study, no statistically significant differences were found. There was a loss of a patient of HIT due to health problems unrelated to the training program.

**TABLE 1:** Baseline characteristics of the sample (mean ± SD).

Parameters	High‐intensity training (*n* = 13)	Low‐intensity training (*n* = 13)	*p*‐value
Age (years)	44.31 ± 12.13	47.31 ± 6.54	0.443
MIP (cm H_2_O)	20.38 ± 6.62	21.38 ± 4.84	0.664
MEP (cm H_2_O)	25.08 ± 5.82	25.46 ± 5.07	0.859
FEV_1_ (liters)	1.26 ± 0.34	1.25 ± 0.24	0.958
PEF (L/min)	69.23 ± 19.69	64.23 ± 15.55	0.480

Abbreviations: cm H_2_O, centimeters of water; FEV1, forced expiratory volume in 1 s; MIP, maximum inspiratory pressure; MEP, maximum expiratory pressure; PEF, peak expiratory flow.

Pre‐ and postintervention outcome comparisons between groups are represented in Table 2. Initial values, final values, and the difference between pre‐ and postintervention were statistically analyzed. All evaluated variables showed very low initial values compared with the reference values for the healthy population. MIP was 19% of normal values for healthy populations, MEP was 13%, FEV_1_ was 56%, and PEF was 21% (Black & Hyatt, 1969).

**TABLE 2:** Pre‐ and postintervention primary outcomes comparisons between HIT and LIT (mean ± SD).

	High‐intensity training (*n* = 13)				Low‐intensity training (*n* = 13)				*p*‐value between groups
Parameter	Pre	Post	Improvement	*p*‐value	Pre	Post	Improvement	*p*‐value	
MIP (cmH_2_O)	20.38 ± 6.62	28.77 ± 9.03	8.38 ± 3.86	0.000	21.38 ± 4.84	25.85 ± 5.62	4.46 ± 2.96	0.000	0.008
MEP (cmH_2_O)	25.08 ± 5.82	30.85 ± 6.55	5.76 ± 2.45	0.000	25.46 ± 5.07	27.46 ± 5.22	2.00 ± 1.15	0.000	0.000
FEV_1_ (L)	1.26 ± 0.34	1.37 ± 0.33	0.11 ± 0.09	0.002	1.25 ± 0.24	1.31 ± 0.24	0.04 ± 0.07	0.049	0.082
PEF (L/min)	69.23 ± 19.69	79.08 ± 19.46	9.85 ± 8.1	0.001	64.23 ± 15.55	66.08 ± 15.98	1.85 ± 3.89	0.113	0.005

Abbreviations: MIP, maximum inspiratory pressure; MEP, maximum expiratory pressure; cm H2O, centimeters of water; FEV_1_, forced expiratory volume in 1 s; PEF, peak expiratory flow; Post, outcome measure after intervention; Pre, outcome measure before intervention.

Significant differences were observed in MIP and MEP between pre‐ and posttreatment, with large effect sizes in HIT (*p* = 0.000 and *d* = 2.17, *p* = 0.000 and *d* = 2.35) and LIT (*p* = 0.000 and *d* = 1.51, *p* = 0.000 and *d* = 1.73). FEV1 also showed significant differences, but with moderate effect sizes in the low‐load group, HIT (*p* = 0.002 and *d* = 1.13) and LIT (*p* = 0.049 and *d* = 0.61). PEF only had statistically significant differences in HIT (*p* = 0.001 and *d* = 1.20), while patients in the LIT group increased their PEF without statistical significance.

For adult CP patients, who trained with high loads, HIT, improved all the respiratory parameters evaluated. MIP increased by 29%, MEP by 19%, FEV_1_ by 13%, and PEF by 8%. On the other hand, patients who trained with low loads, LIT, improved all the respiratory parameters but with a lower percentage; MIP 17%, MEP 7%, FEV_1_ 3%, and PEF 4%, the latter without statistically significant differences.

**FIGURE 2:**
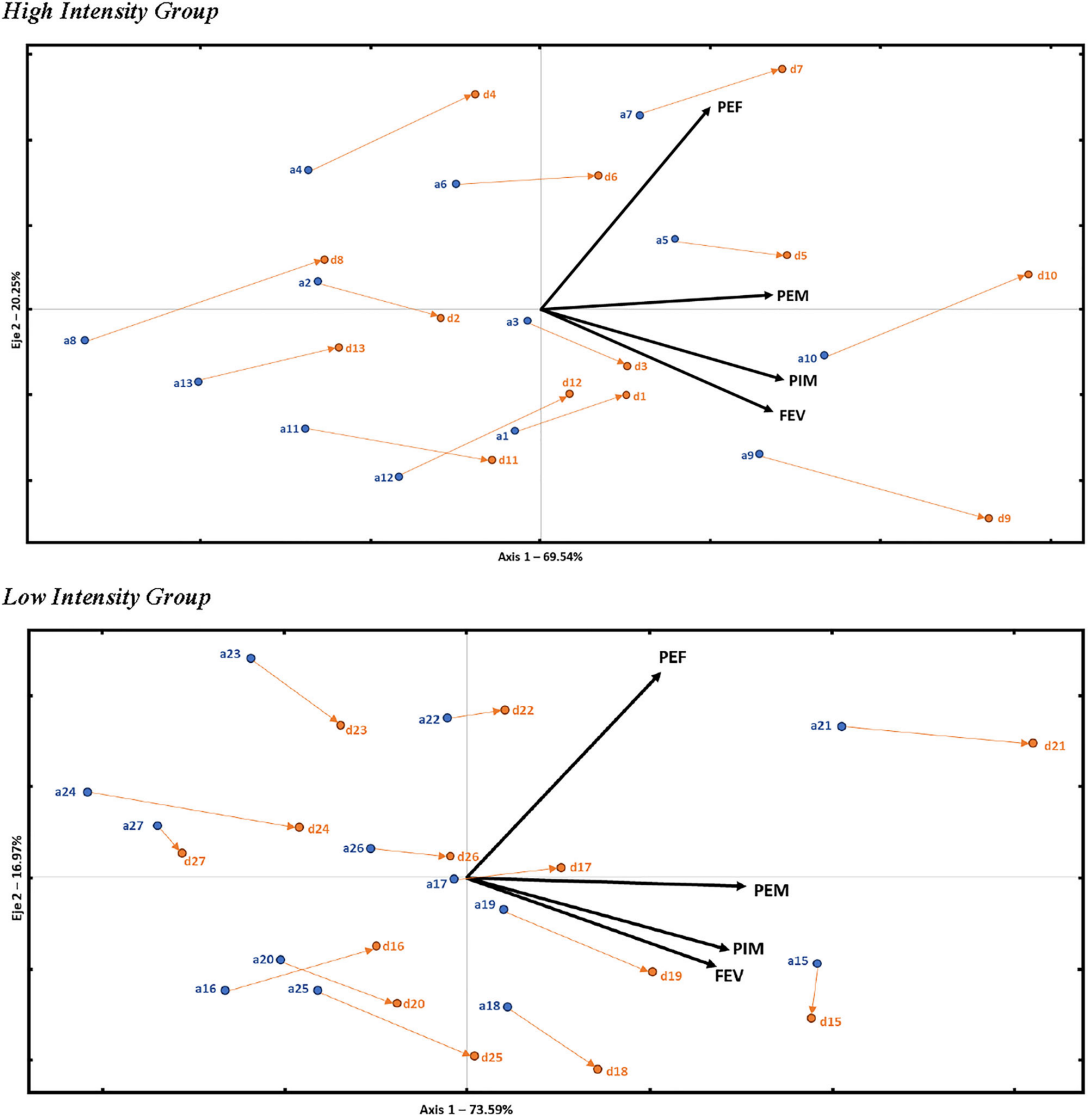
HJ‐Biplot.

Comparing the results between both groups, the high loads achieved stronger improvements in MIP (*p* = 0.008 and *d* = 1.14), MEP (*p* = 0.000 and *d* = 1.96), and PEF (*p* = 0.005 and *d* = 1.25) compared with the low loads, with highly significant results and large effect sizes. FEV1 did not show statistically significant differences between the groups after the training (Figure 2).

The analysis carried out with HJ‐Biplot shows two representations, the first refers to the HIT and the second to the LIT. In each of them, a subspace with the variables MIP, MEP, FEV_1_, and PEF (black vector in the plane) has been built. The position of the patients (points in the plane) is related to the values in those variables. The patients identified with the letter “a” (blue) refer to pretreatment values and the letter “d” (orange) for posttreatment measurements. Also, an arrow for each subject links their position before and after the respiratory treatment.

In this representation, the variables (vectors) are located in the right half‐plane; therefore, the patients positioned further to the right have higher values in all variables. Thus, we can observe that each patient has two locations in the plane, one related to their pretreatment values (blue points) and another corresponding to their posttreatment values (orange points). Most patients experience a horizontal shift (arrows), which translates to an improvement for these patients, which is more noticeable in HIT than in LIT, as their arrows are longer. Additionally, it is worth noting that there are three patients in the LIT group who have improved very slightly (patients 22 and 27) or have not achieved any improvement (patient 15).

Through this representation, it is observed that there is an improvement in the study patients, more notable in HIT than in LIT.

## 4 DISCUSSION

This study represents the first randomized controlled trial investigating the effects of a supervised IMT program specifically focused on adults with CP. Our findings indicate that an 8‐week IMT program leads to significant improvements in respiratory parameters, demonstrating enhanced respiratory strength and lung function among the participants. The comparison of two distinct training program intensities revealed that higher loads yielded superior outcomes compared with lower loads in this population.

The analyzed sample consisted of institutionalized adults with CP, with an average age of 46 years. This cohort presented considerable respiratory limitations, with baseline values considerably lower than the expected reference values for a healthy population. Training sessions were categorized based on intensity: the “High Intensity Training group” (HIT), which trained at 40% of MIP, and the “Low Intensity Training group” (LIT), which trained at 20% MIP.

The topic of life expectancy in individuals with CP remains contentious, with estimates varying widely. Existing literature suggests a life expectancy range of 30 to 60 years (ASPACE, 2020; Birth Injury Center [Internet], 2022), with some studies indicating that 22% of individuals with CP live to or beyond 58 years (Blair et al., 2019). Consequently, our research sample can be classified as both adults and elderly individuals with CP.

### 4.1 Effects of IMT on respiratory muscle strength

Results demonstrated that respiratory muscle strength, as indicated by MIP and MEP, increased in both training groups, with MIP showing the most significant improvement. Notably, participants in the HIT group achieved greater gains in MIP, aligning with findings from prior studies that examined respiratory strength in individuals with CP (Busta et al., 2022; Lee et al., 2014; Varol‐Kepenek et al., 2021). Alongside MIP, improvements were also observed in MEP, with high‐intensity training outpacing low‐intensity training in this regard. These enhancements can be attributed to the learning effect resulting from repeated exposure to training stimuli, which facilitates better neuromuscular recruitment patterns (Kamen, 2005).

The extent of the improvement in MIP following training in prior studies varied widely: some reported modest gains of around 10% (Anand & Karthikbabu, 2021), while others noted an increase of up to 45% (El‐Refaey et al., 2017; Keles et al., 2018; Varol‐Kepenek et al., 2021). The variability in improvements for MEP was similarly pronounced, with increases ranging from as little as 5% (Anand & Karthikbabu, 2021) to as high as 55% (El‐Refaey et al., 2017; Keles et al., 2018; Varol‐Kepenek et al., 2021). Our findings support the efficacy of high loads (40% MIP) as a more effective methodology in respiratory muscle training for adults with CP, yielding greater enhancements in both MIP and MEP compared with lower loads (29% vs. 17% for MIP and 19% vs. 7% for MEP). The previously tested training loads of around 30% MIP yielded inconsistent results, highlighting the novelty and significance of our comparative analysis of training intensities in this population.

Emerging studies in various populations corroborate our conclusions, consistently suggesting that higher training loads yield superior improvements in respiratory muscle strength (Martin‐Sanchez et al., 2021; Martin‐Sanchez et al., 2020; Menezes et al., 2017; Parreiras et al., 2019).

Regardless of the training intensity, IMT has been postulated as an effective respiratory treatment in people with CP, as has been demonstrated in previous studies (Bhuvaneshwari & Suriliraj, 2021; Busta et al., 2022; Keles et al., 2018; Lee et al., 2014; Varol‐Kepenek et al., 2021).

### 4.2 Pulmonary function

In assessing pulmonary function through FEV1 and PEF, both metrics showed enhancement following IMT; notably, improvements were more pronounced in the high‐intensity group. Specifically, PEF rose by 13% in HIT compared with just 3% in LIT, underscoring the effectiveness of higher training loads. While there were no statistically significant differences in FEV1 improvements between groups, the HIT group still demonstrated greater gains (8%) when contrasted with the LIT group (4%).

These findings indicate that IMT not only bolsters respiratory muscle strength but also positively impacts overall pulmonary function. Our results align with previous research indicating that individuals with CP can benefit from IMT (de Lima Crispim et al., 2022; El Banna et al., 2020; El‐Refaey et al., 2017; Menezes et al., 2017; Parreiras et al., 2019), with many studies utilizing a standard training load of approximately 30% MIP. Previous literature presents a diverse range of outcomes related to FEV1 improvements, from negligible changes (Anand & Karthikbabu, 2021; Black & Hyatt, 1969; Varol‐Kepenek et al., 2021) to an upward increase of 35% (Atia & Tharwat, 2021; Choi et al., 2016; Lee et al., 2014). In contrast, PEF improvements tend to be more uniform, with most studies reporting about a 10% increase (Anand & Karthikbabu, 2021; Atia & Tharwat, 2021; Black & Hyatt, 1969; Choi et al., 2016; Lee et al., 2014; Varol‐Kepenek et al., 2021).

Moreover, the positive effects of IMT on pulmonary function have been evidenced not only in individuals with neurological disorders but also in elderly populations devoid of such pathologies (Kamen, 2005; Martin‐Sanchez et al., 2021; Seixas et al., 2020). This reinforces the generalizability of IMT as an effective intervention.

In conclusion, the current study substantiates the notion that IMT is a beneficial treatment modality for enhancing respiratory parameters in adults with CP. While recent investigations indicate statistically significant improvements post‐IMT, a lack of consensus regarding optimal training parameters—including frequency, intensity, and type—prevails in the literature (de Lima Crispim et al., 2022). Future research is warranted to refine these training protocols further and enhance our understanding of the most effective strategies for this population.

### 4.3 Clinical implications

Current scientific evidence supports the use of IMT in people with CP. However, clinical trials in adult people with CP are nonexistent and there is no established protocol for this population group. Respiratory problems and limitations suffered by elderly people with CP represent a challenge for respiratory physiotherapy today. This study will increase evidence knowledge in this matter and lay the foundations for the application of a specific IMT protocol in adult people with CP.

### 4.4 Trial limitations and recommendations

The heterogeneity of adult individuals with CP, implementing stratified randomization based on GMFCS levels to create study arms would have prevented this limitation. The difficulty in recruiting the sample, the small number of adult people with CP and the time available to carry out the study did not allow it to be done.

The participants from the province where the study was conducted may not represent the total population.

The inclusion of a third group, with the same characteristics as our sample, which did not train with any respiratory dispositive, as a control group, to contrast results.

An important limitation is the time of intervention, as there are no other similar studies in this population group, we had to follow the IMT guidelines for other groups. However, as with most exercise protocols, understanding the “acute” effects of IMT in other population groups, may ensure better programming of chronic protocols and increase our understanding of the possible benefits and side effects.

The lack of follow‐up of the volunteers in the subsequent months after the training did not allow us to evaluate the long‐term efficacy of IMT.

Future research that aims to investigate the benefits in institutionalized cerebral palsy patients obtained with IMT should evaluate the quality of life, trunk control, or dyspnea to achieve a global vision of the effects. Follow‐up of the volunteers for 6 months would be interesting to meet the efficacy of IMT once the participants stopped using it.

This is the first respiratory study of its kind carried out in adults with cerebral palsy we want to lay the foundations to continue with research in the elderly population with cerebral palsy due to the increasing life expectancy.

## 5 CONCLUSION

This study showed that an 8‐week protocol with IMT improved respiratory muscle strength and pulmonary function in adult people with CP. Training with a 40% MIP load improved all the evaluated parameters and was the most effective treatment for adult people with CP.

## AUTHOR CONTRIBUTIONS

Carlos Martin‐Sanchez: Conceptualization; methodology; investigation; supervision; project administration; writing―original draft; writing―review and editing. Fausto Jose Barbero‐Iglesias: Investigation; project administration; visualization. Victor Amor‐Esteban: Software; data curation; formal analysis. Marta Martin‐Sanchez: Investigation; writing―review and editing; supervision. Ana Maria Martin‐Nogueras: Writing―original draft; resources; funding acquisition; validation.

## CONFLICT OF INTEREST STATEMENT

The remaining authors declare no conflict of interest.

### PEER REVIEW

The peer review history for this article is available at https://publons.com/publon/10.1002/brb3.70044

## PATIENT CONSENT STATEMENT

All subjects have signed the informed consent before their inclusion in the study.

## PERMISSION TO REPRODUCE MATERIAL FROM OTHER SOURCES

Available.

## CLINICAL TRIAL REGISTRATION

The study was registered in the clinical trials database of the United States National Library of Medicine (www.clinicaltrials.gov) with the number of registration NCT04915170.

## Data Availability

Further inquiries can be directed to the corresponding author. All the data collected in the study will be shared. Individual deidentified participant data are available. Additional and related documents such as study protocol or statistical analysis are available. The data will be available from January 2024 for as long as necessary. The data will always be shared with the approval of the corresponding author after analyzing the purpose they are to be used.
